# Recent advances in the application of ionic liquids in antimicrobial material for air disinfection and sterilization

**DOI:** 10.3389/fcimb.2023.1186117

**Published:** 2023-05-17

**Authors:** Xizi Song, Rujin Tian, Kai Liu

**Affiliations:** ^1^Division of Environment and Sustainability, Hong Kong University of Science and Technology, Clear Water Bay, Hong Kong, Hong Kong SAR, China; ^2^University of Health and Rehabilitation Sciences Qingdao Hospital (Qingdao Municipal Hospital), Qingdao, Shandong, China

**Keywords:** antimicrobial ionic liquids, dicationic ionic liquids, polymer ionic liquids, antimicrobial mechanism, air sterilization

## Abstract

Airborne transmission is one of the most unpredictable routes of infection. Nowadays, airborne diseases increase ever than before because of the complex living air environment. Apart from the inorganic particles, active microorganisms including bacteria, viruses, and fungi are incorporated in the pathogens acting as threaten to public health, which can hardly be treated by the traditional air purification methods based on adsorption. Therefore, effective filtration material with antimicrobial activity is demanded to solve the problem. Ionic liquids (ILs) are a category of salts that remain liquid at room temperature. The stable physico-chemical properties and extremely low vapor pressure make them suitable for a wide range of applications. Thanks to the numerous combinations of cations and anions, as well as the ability of inheriting properties from the parent ions, Ils are believed to be a promising industrial material. In recent decades, several Ils, such as imidazolium, pyridinium, pyrrolidinium, phosphonium, and choline, have been found to have antimicrobial activity in their monomeric or polymeric forms. This work focuses on the antimicrobial activity and safety of the latest types of ionic liquids, discussing the synthesis or manufacturing methods of Ils for air purification and filtration. Furthermore, possible applications of Ils antimicrobial materials in medical instruments and indoor environments are mentioned to encourage the scientific community to further explore the potential applications of Ils.

## 1 Introduction

**Figure 1:**
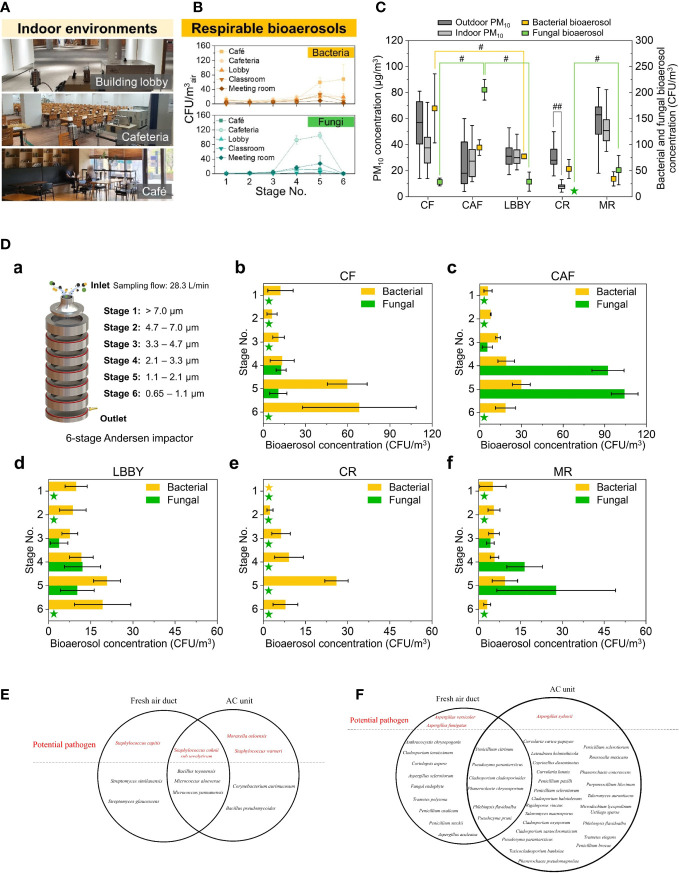
**(A)** Sampling location; **(B)** Concentration of Respirable bioaerosols in 5 locations; **(C)** Total bioaerosols and PM10 concentrations at the sampling locations. For outdoor and indoor PM10 concentrations, boxes indicate the 25th–70th percentiles; lines inside the boxes indicate the median, and whiskers represent the maximum and minimum values. For bacterial and fungal bioaerosol concentrations, error bars represent the standard deviation. #p < 0.05 and ##p < 0.01, respectively. Asterisks indicate no colony formation; **(D)** Bacterial and fungal bioaerosol concentration by size. (a) Schematic of the six-stage Andersen cascade impactor. (b–f) Bioaerosol concentrations for each stage and sampling location. Asterisks indicate no colony formation. Error bars represent the standard deviation. Reproduced with permission from Elsevier Ltd. (Jeong et al., 2022, 100182); **(E)** Identified bacterial and **(F)** fungal species emanated from the Air Conditioning and Mechanical Ventilation System. Reproduced with permission from (Xiong et al., 2020, 762–75).

The concept of airborne transmission, also known as aerosol transmission, has gained prominence in recent decades due to frequent outbreaks of epidemic diseases. Unlike the traditional understanding of air pollution caused by inorganic particles or noxious gases, the study of aerosols investigates the effect of bioactive or infectious particles such as viruses, bacteria, or fungi, which can be suspended in air for not only a short distance as droplets, but also a long-range travel as air mass and have a strong influence on respiratory system (**Figure 1**) (Duchaine and Roy, 2022, 154117; Mutuku et al., 2020, 1172–96; Morawska et al., 2020, 105832; Jeong et al., 2022, 100182; Xiong et al., 2020, 762–75). With modern lifestyles resulting in an ever-increasing amount of time spent indoors and the unavoidable production of aerosols from breathing, talking, coughing, flushing toilets, etc., the best way to protect the public from infectious aerosol is to sterilize the air through the daily use of heating, ventilation, and air conditioning (HVAC) systems. Additionally, personal protective devices such as masks or atomizers with antimicrobial and disinfection components are sought after for individual prevention.

Unfortunately, most commercial HVAC systems and portable devices are not equipped with disinfection capabilities. Furthermore, the accumulation of dust containing bioactive pathogens can create a source of contamination, thus increasing the risk of infection (Xiong et al., 2020, 762–75). Portable devices can be designed to be disposable, but the short lifespan and the health risks of respiratory irritation posed by antimicrobial agents, such as ozone and chlorides, currently impede their widespread use (Bruchard et al., 2021, A35D-1662; J. (Jim) Zhang et al., 2019, vol. 10).

Ionic liquids (ILs) are a category of highly tunable salts that retain the properties of their parent ions. Unlike the strong electrostatic forces between ions in normal ionic crystals, the ionic interaction is ILs is frail owing to the structural heterogeneity of sterically hindered asymmetric coordination complexes, disturbing the formation of an intensive crystal, which consequently leads to the extremely low melting point of ILs. In 1951, Frank H. Hurley and Thomas P. Wler Jr. discovered a mixture of ethyl pyridinium bromide and aluminum chloride that had a very low melting eutectic (-40°C) at 67-mole percent aluminum chloride, which was used to prepare electrode as an intermediate for metal coating/deposition (Hurley and WIer, 1951, 203). The eutectic mixture was then believed to be a coordination complex of aluminum and pyridinium bromide. Following this discovery, similar compounds with low melting points but high electroconductivity were reported in various fields, such as electrochemistry, synthesis and catalysis, and biomedicine. These compounds are eventually known as “Ionic liquids”. Rather than being a new class of material, ILs are more like an innovative concept of material synthesis, directing the industry in a more function-oriented way. Since the quaternary ammonium cation has a strong bactericidal effect, and its coordination structure can form ILs to reduce its toxicity, much attention has been given to the application of ILs in antibacterial materials, led by quaternary ammonium ILs. As an increasing number of antimicrobial ILs have been revealed, research has extended further in exploring ways for their application. In this study, we discuss the antimicrobial activity and safety of the latest types of ionic liquids and manufacturing methods of ILs for air purification and filtration. Furthermore, possible applications of ILs antimicrobial materials in medical instruments and indoor environments are mentioned to encourage the scientific community to further explore the potential applications of ILs.

## 2 Application of ILs with antimicrobial activity

Antimicrobial activity has been one of the most intensively studied properties of ILs. In recent decades, several ILs such as imidazolium, pyridinium, pyrrolidinium, phosphonium, and choline have been shown to have antimicrobial activity in their monomeric or polymeric forms (Gilmore, 2011; Siopa et al., 2016, 5909–16; Ibsen et al., 2018, 2370–79). According to the application form and synthesis method, antimicrobial ILs can be roughly divided into monomeric ILs, dicationic ILs, and polymeric ILs.

### 2.1 Monomeric ILs with antimicrobial activity

The history of RTILs can be originated in 1914, when ethylammonium nitrate was found on the upper layer after mixing concentrated nitric acid and ethylamine (Ghosh, 2021, 241–44), which inspired the mainstream idea of ILs preparation by combining cations based on Lewis acid-base reactions or anion metathesis (Singh and Savoy, 2020, 112038). Conventional methods for cationic precursor preparation involves protonating amines with acid or quaternizing amines with haloalkanes, while anionic precursors can be prepared by treating halide salts with Lewis acids (D. Fang et al., 2008, 108–11; Ratti, 2014, 1–16). As an example, N-alkylated commercial chemicals with certain functional head groups are a common method for forming target cation precursors, and monomeric ILs can be prepared through electrophilic reactions between the precursors and terminal haloalkanes (Gilmore et al., 2013, 873–76; Florio et al., 2019, 109907).

Considering the convenience of introducing diverse types of side chains on cationic head groups during synthesis, the antimicrobial potency of earlier generations of ILs is mainly attributed to their cationic moiety. Imidazolium is one of the most industrialized ILs; its antimicrobial effectiveness has been demonstrated against bacteria, fungi, and viruses. Similarly, extensive research has reported cationic ILs with confirmed antimicrobial potency against microorganisms (Docherty and Kulpa, 2005, 185–89; Nikfarjam et al., 2021, 2104148).

Contact sterilization is the main bactericidal route for most monomeric or DILs, restricting the application of ILs in bactericidal materials to the scope centered on antibacterial additives. Coating is an optimal way of forming a concentrated IL surface for improved bactericidal performance (Fallah et al., 2021, 102454). However, ILs exhibit stable physiochemical properties and their normal viscosity ranges from 10 to 2000 cP, which brings difficulties in surface spreading and fixation (Zhou et al., 2014, 275–307).

Inspired by its role as an outstanding solvent, alcohol, acetonitrile, and tetrahydrofuran were used to prepare ILs solutions, increasing their viscosity and making them easily spreadable on both porous and non-porous surfaces to assemble an antimicrobial layer. The organic solvents can then be removed using a vacuum pump or heat (Yeung et al., 2020).

**Figure 2:**
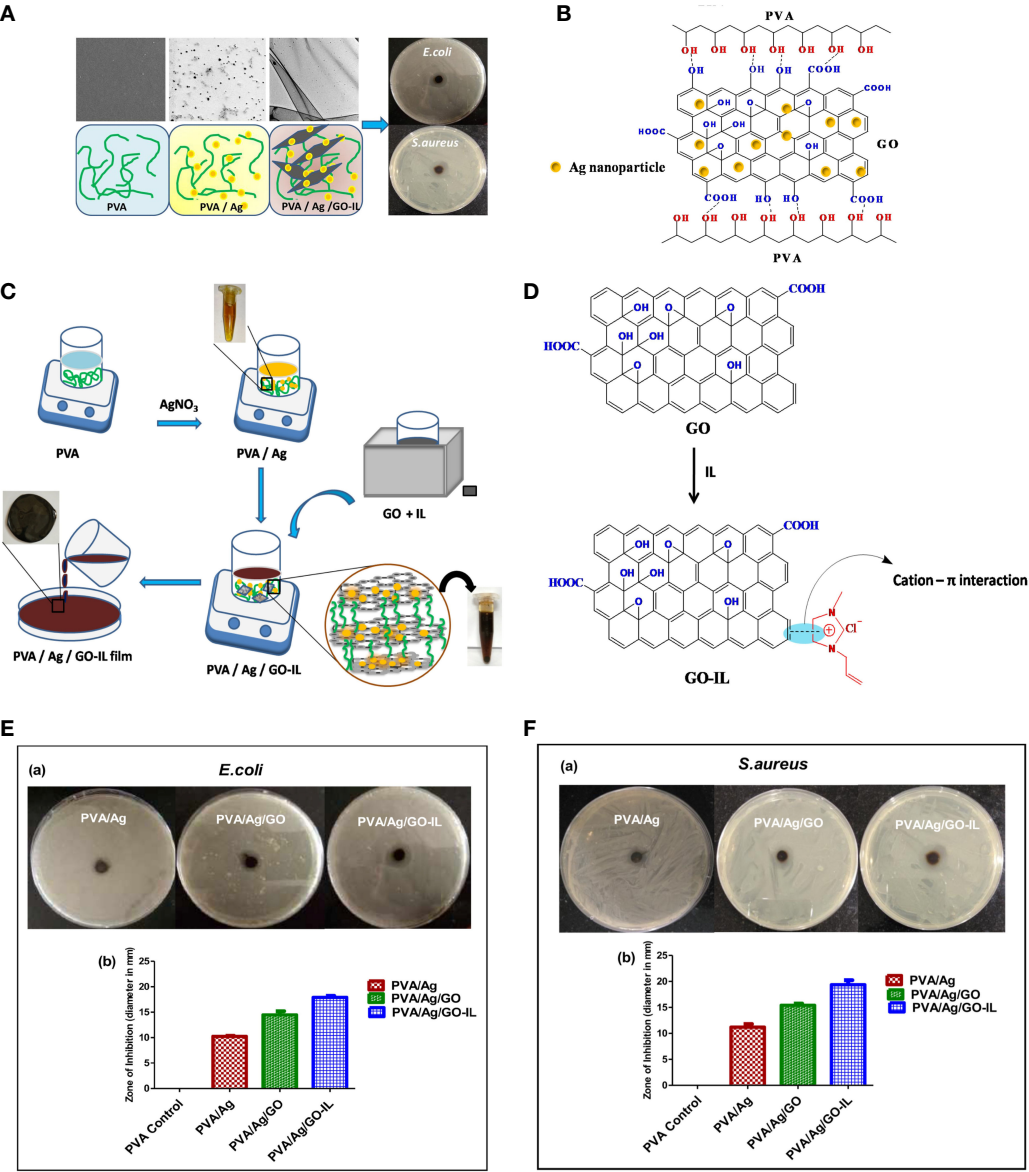
**(A)** Illustration of Ionic liquid-assisted fabrication of poly(vinyl alcohol)/nanosilver/graphene oxide (GO) composites and their cytotoxicity/antimicrobial activity; **(B)** Schematic presentation of interaction between PVA and GO in PVA/Ag/GO; **(C)** Schematic presentation of the preparation of PVA/Ag/GO-IL nanocomposite film; **(D)** Schematic cation-π interaction between IL and graphene oxide; (**E**, a) Antibacterial activities of PVA/Ag, PVA/Ag/GO and PVA/Ag/GO-IL against E coli (b) Graphical representation for zone of inhibition of nanocomposite films against E coli; (**F**, a) Antibacterial activities of PVA/Ag, PVA/Ag/GO and PVA/Ag/GO-IL against S. aureus (b) Graphical representation for zone of inhibition of nanocomposite films against S. aureus. Reproduced with permission from (Sahu et al., 2021, 124524).

**Figure 3:**
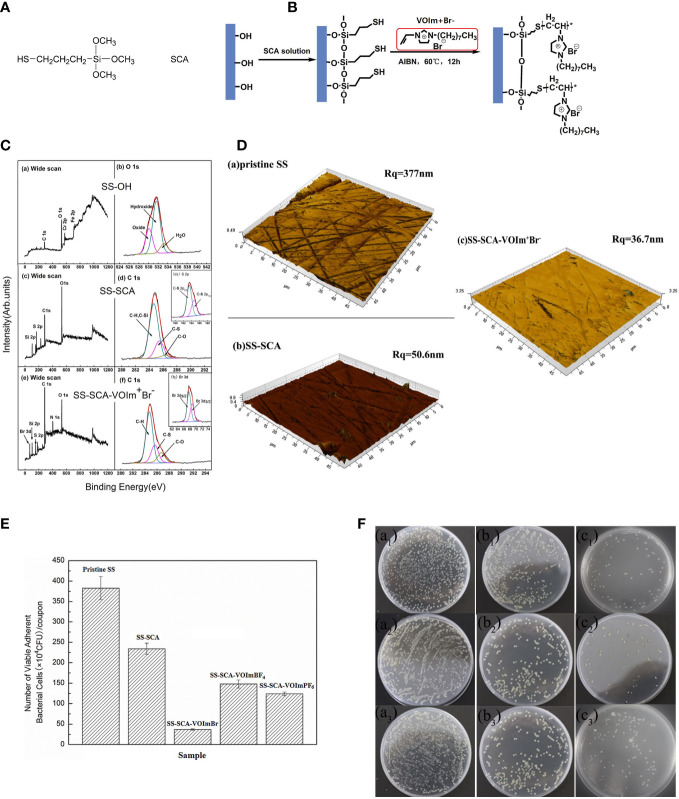
**(A)** The chemical structure of (3-mercaptopropyl)trimethoxysilane (SCA); **(B)** Schematic diagram illustrating the process of coupling SCA to hydroxyl stainless steel (SS) surface, and the grafting of the coating IL; **(C)** Wide scan and C1s core-level spectra of **(A, B)** the SS OH surface, and **(C, D)** the SS-SCA surface, **(E, F)** SS-SCA-VOIm^+^Br^−^ surface. Insets of (d1) and (f1) corresponding to the S2p and Br3d core-level spectra of the SS-SCA and SS-SCA- VOIm^+^Br^−^ surfaces, respectively; **(D)** AFM images of the **(A)** pristine stainless steel **(B)** SS-SCA **(C)** SS-SCA- VOIm^+^Br^−^; **(E)** Number of viable adherent cells of E coli (CFU per coupon) on the pristine SS, SS-SCA, SS- VOIm^+^Br^−^, SS-VOIm+BF4^−^ and SS- VOIm^+^PF6^−^ surfaces respectively after incubation with E coli suspension at 37 °C for 12 h The cell number was determined by the spread plate method. Each error bar represents the standard deviation calculated from three replicates; **(F)** Inhibitory effects of the (a) pristine SS, (b) SS-SCA and (c) SS-SCA- VOIm^+^Br^−^ surfaces against E coli after immersion in E coli suspension for 12 h, respectively. Reproduced with permission from (Pang et al., 2015, 162–68).

The fixation of ILs can be solved through various interactions, depending on the coated surface. Graphene oxide, due to the presence of oxygen functionalities on its surface, is negatively charged. Thus, ILs with antimicrobial cations, such as imidazolium, can be anchored to graphene oxide through the formation of cation-π interactions (**Figure 2**) (Sahu et al., 2021, 124524). A hollow polymeric nanosphere composed of 1,4-bisbenzenedimethanol monomer is reported to be embedded with imidazolium-based ionic liquids by the sequential covalent bonding of imidazole and bromoalkene onto the nanosphere (Zhang et al., 2022, 5556–68). A gelatin matrix is able to hydrobond biobased IL, choline salicylate through various bonding sites, resulting in not only microbial growth inhibition, but also a plasticizing effect to form a film for packaging (Mehta and Kumar, 2019, 8631–36). ILs can also be applied to stainless steel by covalently bonding the thiol-functionalized site created by (3-mercaptopropyl)trimethoxysilane silane bonded on stainless steel. (Mehta and Kumar, 2019, 8631–36; Pang et al., 2015, 162–68). (**Figure 3**)

As the variety of ILs expands, the concept of task-specific ILs is being renewed, breaking the boundary of remaining in a liquid state at room temperature while still preserving their distinctive properties as solvents. The concept of antimicrobial ILs is leaning towards incorporating existing biologically active moieties into ILs to create high-performance bactericidal materials. (Gilmore et al., 2013, 873–76).

### 2.2 Dicationic ILs

Among the antimicrobial ILs, cationic moieties are believed to perform stronger bactericidal than anions. Consequently, DILs have emerged, following the trend of attempting to enhance the bactericidal potency. The synthesis of DILs shares the same idea of precursors as monomeric ILs, but with an additional procedure of linking the two cationic moieties through alkane substitution. Dicarboxylate or two anions are involved as counterions (Kuhn et al., 2021, 639; Kuhn et al., 2020, 112983; Gindri et al., 2014, 62594–602; Brunel et al., 2020, 127389).

**Figure 4:**
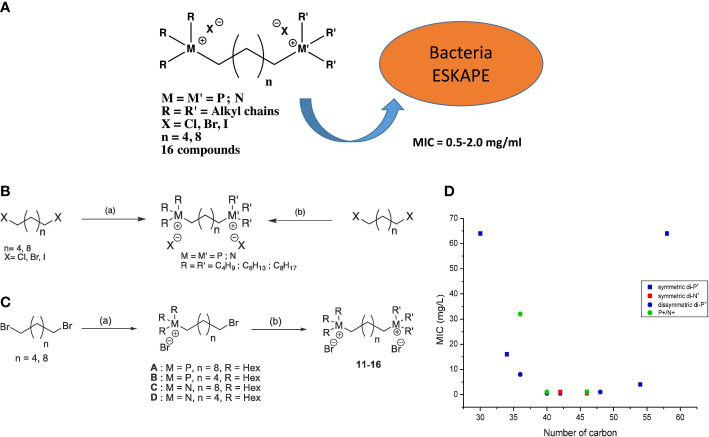
**(A)** Scheme of experimental homodicationic ILs and the conclusion of their antimicrobial performance; **(B)** Scheme of synthetic pathway of homodicationic ILs; **(C)** Scheme of synthetic pathway of homodicationic ILs; **(D)** Effect of the total number of carbons and symmetry of 16 ILs on their bactericidal efficiency for S. aureus. Reproduced with permission from (Brunel et al., 2020, 127389).

According to the cation types, DILs can be divided into homo- and hetero-DILs. A homo-DIL composed of two 1-alkyl-3-methylimidazolium-based ILs has been demonstrated to effectively inhibit the growth of several airborne pathogenic microorganisms, such as Escherichia coli, Enterococcus faecalis, methicillin-resistant S. aureus, methicillin-susceptible Staphylococcus aureus, Klebsiella pneumoniae, Pseudomonas aeruginosa, Acinetobacter baumannii, and Klebsiella aerogenes, as well as yeast and fungi (Kuhn et al., 2021, 639). Quaternary phosphonium and ammonium ions are two well-known coordinate ions found in antimicrobial ionic liquids. Hetero- and homo-dimerized ILs containing these two ions have been proven to exhibit high antimicrobial activity (MIC=0.5 mg/L) against a range of Gram-positive and Gram-negative bacterial strains, including major human pathogens with multi-drug resistance (**Figure 4**) (Brunel et al., 2020, 127389). Differentiated from the alkylation of coordinate ions, the hybridization of pyridinium hydrazone yield DILs with a more complex structure. These DILs display increased resistance to the growth of methicillin-resistant Staphylococcus aureus, Clostridium difficile, Escherichia coli, Neisseria gonorrhea, and Candida albicans due to the enhanced hydrophobicity resulting from the introduction of a terminal phenoxy group (Rezki et al., 2019, 431–44).

Except for the expected enhanced disinfectant activity, another appealing advantage of DILs (Dicationic Ionic Liquids) is their reduced toxicity. Toxicity reduction caused by multiple cationic coordinate complexes was verified by comparison between imimdazolium-based DILs and their monomeric ILs (Gindri et al., 2014, 62594–602). MG Montalbán et al. reported the toxicity assessment of 1-alkyl-3-methylimidazolium-, 1-alkyl-1-methylpyrrolidinium-, and pyridinium-based mono-cationic and dicationic ILs, and all the homo- or hetero- DILs presented lower toxicity than their corresponding monomeric ILs according to the EC50 against luminescent bacterium Vibrio fischeri (Montalbán et al., 2018, 129–35).

### 2.3 Polymeric ILs

As a continuation of the idea of improving antimicrobial properties and reducing toxicity, polymeric ionic liquids (PILs) have attracted great interest. The monomer of PILs comprises three parts: a head, which is usually named after the pristine functional coordinate ion in monomeric ionic liquids; a tail, which is derived from the side chain linked to the coordinate ion; and a spacer or linker, which is the structure connecting the head and forming the polymeric backbone (Vereshchagin et al., 2021, no. 13). This structure solidifies the ILs and significantly reduces their toxicity while improving their bactericidal efficiency compared to the corresponding monomeric form (Qin et al., 2017, 10504–11; Muñoz-Bonilla and Fernández-García, 2018, 135–49). Furthermore, since the ILs with antibacterial activity are used as the base material rather than a coating, the problem of fixing ILs to the substrate material no longer exists, thus allowing more flexibility in their application for antimicrobial purposes.

**Figure 5:**
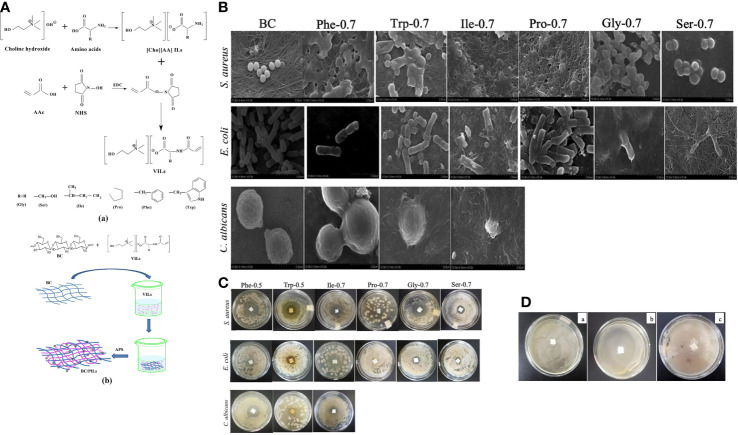
**(A)** Synthetic route of Vinyl ILs and bacterial PILs crosslinked cellulose membrane crosslinked. **(B)** FE-SEM images of microbial cells cultured on bacterial cellulose (BC) and BC/PIL membranes; **(C)** Drug resistance of S. aureus, E coli, and C. albicans to different BC/PIL membranes; **(D)** Inhibition zone of the BC membrane against S. aureus (a), E coli (b), and (c) albicans (c). Reproduced with permission from (He et al., 2020, 14784–96).

Given the adjustable preparation methods and wide range of applications, PILs membranes were first explored. Unlike the commonly used dimerization of two ILs in DIL, the synthesis of PILs may simultaneously form a polymeric backbone with the production of ILs. Pyrrolidinium-based PIL polymers were prepared by mixing and heating diallyl methyl ammonium hydrochloride and (S)-2-(ethyl propionate)-(o-ethyl xanthate) through a typical reversible addition-fragmentation chain transfer polymerization (Muñoz-Bonilla and Fernández-García, 2018, 135–49). In contrast to polyethylene terephthalate membrane, the prepared PIL membrane comprising 1-butyl-methyl pyridinium demonstrated a high inhibition rate of over 95% in 4 hours of contact with Escherichia coli and Staphylococcus aureus. The successful production of a copolymer membrane of poly(1-vinyl-3-butylimidazolium bromide) (VBIMBr) and polyvinyl alcohol (PVA), as a representative, indicates the feasibility of hybridizing the PIL and existing plastic materials with excellent mechanical properties (H. Fang et al., 2019, 153–64). The hydrogen bond formed between the hydroxyl group of PVA and the primary amine of VBIMBr crosslinked the polymer chains and produced an intense membrane with bactericidal potency against airborne microbes. In addition to polycationic PILs, IL can also be polymerized in an anionic form; the copolymer of bacterial polysaccharide cellulose and ammonium-based poly-vinyl ILs were crosslinked to form a soft transparent membrane while retaining their intrinsic antimicrobial activity against Staphylococcus aureus, Escherichia coli, and Candida albicans (He et al., 2020, 14784–96) (**Figure 5**).

Nanofiber-based membranes composed of PILs have been found to possess more practical merits than simply being used as antimicrobial compositions, making them ideal components for developing healthy, portable antimicrobial devices. A PIL-based multilayer nanofiber was prepared by electrospinning of 1-hexyl-3-vinylimidazole bis(trifluoromethylsulfonyl) imide (HVIm-TFSI) and N-(2-(acryloyloxy)ethyl)-N,N-dimethylhexan-1-aminium bromide (ADHex-Br) (Zheng et al., 2022, 2106570). The membrane consists of two sides, with the HVIm-TFSI acting as the hydrophobic side due to the presence of the connected counterion bis(trifluoromethanesulphonyl) imide, and the ADHex-Br comprised hydrophilic side allowing moisture to contact the hydrophobic side. This PIL-based porous material has the capacity to be bactericidal when exposed to moist airflows.

## 3 Conflict between antimicrobial mechanism and toxicity of ILs

The interactions between cationic moieties of ILs and the cell membrane may explain the antimicrobial activity of these ILs. Similar to cationic biocides, the hydrophobic contact with the outer phospholipid of the cell membrane causes a structural transformation of the lipid bilayer through electrostatic interactions with membrane proteins, inserting the cationic head group into the hydrophobic area of the membrane and destroying its integrity. This disruption of the cell membrane then leads to the leakage of intracellular cytoplasm and further promotes the disruption of the cell wall. For example, imidazolium-based ILs interact with the cell membrane in a manner similar to surfactants. As a result, the highly polar imidazolium ring strongly interacts with the phospholipid head, remaining near the interface between water and the membrane. The cation then has a preference for orienting its alkyl side chain into the hydrophobic region composed of phospholipid tail groups, with the long alkyl chains usually diving deeper into the hydrophobic regime (Yoo et al., 2014, 8641–51).

Apart from the potential totoxicity caused by IL-membrane interaction, suppressing or inhibiting the generation of biofilm has greater importance in the antimicrobial field. Except for microbial cells, the primary composition of the extracellular polymeric substance of biofilm is polysaccharides (Donlan, 2002, 881–90). The cross-linked saccharide chains hold the individual cells together and finally form a matrix, increasing the survival of bacterial cells under demanding environments and thus bringing more difficulties for sterilization and purification.

The most concerning adverse effect of applications based on ILs is their toxicity. Considering the IL-membrane interaction and the similar membrane structure of bacterial and mammalian cells, ILs with outstanding antibacterial performance can hardly be considered as positively devoid of cytotoxicity. Therefore, the balance between the antimicrobial activity and the safety of ILs has been a focus of attention.

Three factors are typically mentioned to profile the biological activity of ILs: (1) the side chain of the cation; (2) the functional group (or head group) of the cation; (3) the anion. The long alkyl side chain connected to the cation head group gives the IL an intricate spatial structure and, to some extent, reduces the polarity of the cation. The widely accepted “side chain effect” illustrates the trend of increased toxicity, as well as bactericidal activity, with an elongated alkyl side chain, which is applicable to both monomeric and polymeric ILs (Łuczak et al., 2010, 593–601; Kuhn et al., 2020, 112983). The reduction in surface tension of the aqueous solutions caused by the micelle formation of ILs has been offered as one explanation for the “side chain effect” (Łuczak et al., 2010, 593–601). Moreover, the increasing lipophilicity resulting from the elongation of the aliphatic chain also contributes to severe membrane interaction. Consequently, introducing polar functional groups on the side chain decreases the lipophilicity of the cation, thus reducing the toxicity of ILs (Stolte et al., 2007, 1170–79).

Imidazolium, pyrrolidinium, ammonium, morpholinium, benzimidazolium, and piperidinium are commonly used head groups, and their interaction with IL membranes has been compared. It has been shown that ILs with unshielded charges, such as benzimidazolium, imidazolium, and ammonium, cause strong reorientation of the phosphocholine moiety in the lipid head group of the membrane, and exacerbate the quivering of the side chain of the lipid chain in the membrane. This is most pronounced for large cations such as benzimidazolium and piperidinium. Surprisingly, the relatively polar headgroup of morpholinium has no observed effect on the bilayer (Kaur et al., 2021, 954–63). A similar trend has been summarized and reported in terms of decreasing toxicity against Daphnia magna: phosphonium > ammonium > imidazolium > pyridinium > morpholinium > pyrrolidinium > thiophenium. (Hossain et al., 2011, 990–94).

The anion effect is usually not as dramatic as that of cations, since no distinct trend can be concluded for the anion structure which often varies in the experimental system and anions (Stolte et al., 2007, 1170–79). However, revelations have been provided for the antimicrobial activity or toxicity of reported anions. For instance, increasing antifungal activity was found in ILs with identical short-chain cations on Candida albicans, following the order of anions: bis(trifluoromethylsulfonyl)imide > octyl sulphate ≈ tetrafluorobromide > methyl sulphate > chloride > trifluoromethyl aryl sulfonate (Jalal et al., 2014, 260–69); methylchlorophenoxypropionic acid and dicamba demonstrated lower toxicity than their bromide counterparts (Piotrowska et al., 2017, 114–19); a toxicity decrease follows the order trichloroacetic acid > thiocyanate ≈ dicyanamide ≫ chloride > methylsulfate (Mester et al., 2015, 96–101).

Three adjustments can be applied to subside the toxicity of ILs: (1) introduction of polar functional groups in the side chain; (2) addition of low-toxic cationic coordinate complexes; (3) decrease of alkyl chain length. Considering the reorientation of ILs on the membrane-water interface, it is hypothesized that the reduction of ILs’ toxicity could be explained as the polarity hampering the stretching of the side chain, thus delaying the breakdown of the cell membrane.

## 4 Conclusion

Antimicrobial IL is capable of utilizing various material synthesis methods to process or combine the proved effective bactericidal groups to cater to different needs of air sterilization. This article describes the three basal forms of antimicrobial IL and the representative novel antimicrobial ILs within each form. Based on the effective bactericidal groups, each antimicrobial IL has different preferences in terms of killing microorganisms. For instance, imidazolium ILs have a slightly stronger inhibitory effect on Gram-positive bacteria than Gram-negative bacteria. Similarly, due to the wide range of structures and forms of antimicrobial IL, the cytotoxicity can only be predicted based on the basal moieties as cation or anion functional group. Overall, the form of PIL can reduce the toxicity of the original antimicrobial IL to a great extent. Furthermore, based on experimental results, several common components of antimicrobial IL were sorted addressing their toxicity to provide useful references for developing and applying new antimicrobial IL. Conversely, IL and DIL can be used as additives or coatings to modify the existing air filter apparatus, endowing them with high-performance bactericidal functions. Generally, developing antibacterial ILs provides a promising solution to the challenge of air quality and epidemic prevention. With the right preparation methods and material combinations, antibacterial ILs can be applied to a wide range of scenarios, making them a crucial part of the fight against air pollution and the spread of diseases.

## Author contributions

LK contributed to the conception and design of the study. SX organized the database. SX and TR performed the statistical analysis. SX wrote the first draft of the manuscript. LK, SX, and TR wrote sections of the manuscript. All authors contributed to manuscript revision, read, and approved the submitted version.

## References

[B1] Bruchard W. Bajracharya A. Johnston N. (2021). “Volatile organic compounds from covid-19 disinfectants - effects on indoor air quality,” in 2021:A35D-1662. AGU Fall Meeting Abstracts 2021. New Orleans, LA

[B2] Brunel F. Lautard C. Garzino F. Raimundo J.-M. Bolla J.-M. Camplo M. (2020). Phosphonium-Ammonium-Based di-cationic ionic liquids as antibacterial over the ESKAPE group. Bioorgan. med. Chem. Lett. 30 (18), 127389. doi: 10.1016/j.bmcl.2020.127389 32717610

[B3] Docherty K. M. Kulpa C. F. Jr. (2005). Toxicity and antimicrobial activity of imidazolium and pyridinium ionic liquids. Green Chem. 7 (4), 185–189. doi: 10.1039/b419172b

[B4] Donlan R. M. (2002). Biofilms: microbial life on surfaces. Emerg. Infect. Dis. 8 (9), 881–890. doi: 10.3201/eid0809.020063 12194761 PMC2732559

[B5] Duchaine C. Roy C. J. (2022). Bioaerosols and airborne transmission: integrating biological complexity into our perspective. Sci. Total Environ. 825, 154117. doi: 10.1016/j.scitotenv.2022.154117 35218821 PMC8865953

[B6] Fallah Z. Zare E. N. Khan M. A. Iftekhar S. Ghomi M. Sharifi E.. (2021). Ionic liquid-based antimicrobial materials for water treatment, air filtration, food packaging and anticorrosion coatings. Adv. Colloid Interface Sci. 294, 102454. doi: 10.1016/j.cis.2021.102454 34102390

[B7] Fang D. Cheng J. Gong K. Shi Q.-R. Zhou X.-L. Liu Z.-L. (2008). A green and novel procedure for the preparation of ionic liquid. J. Fluorine Chem. 129 (2), 108–111. doi: 10.1016/j.jfluchem.2007.09.004

[B8] Fang H. Wang J. Li L. Xu L. Wu Y. Wang Y.. (2019). A novel high-strength Poly(Ionic Liquid)/PVA hydrogel dressing for antibacterial applications. Chem. Eng. J. 365, 153–164. doi: 10.1016/j.cej.2019.02.030

[B9] Florio W. Becherini S. D’Andrea F. Lupetti A. Chiappe C. Guazzelli L. (2019). Comparative evaluation of antimicrobial activity of different types of ionic liquids. Mater. Sci. Eng. 104, 109907. doi: 10.1016/j.msec.2019.109907 31499958

[B10] Ghosh R. (2021). Discovery of room temperature ionic liquid. Resonance 26 (2), 241–244. doi: 10.1007/s12045-021-1122-3

[B11] Gilmore B. F. (2011). Antimicrobial ionic liquids (UK: INTECH Open Access Publisher London).

[B12] Gilmore B. F. Andrews G. P. Borberly G. Earle M. J. Gilea M. A. Gorman S. P.. (2013). Enhanced antimicrobial activities of 1-Alkyl-3-Methyl imidazolium ionic liquids based on silver or copper containing anions. New J. Chem. 37 (4), 873–876. doi: 10.1039/c3nj40759d

[B13] Gindri I. M. Siddiqui D. A. Bhardwaj P. Rodriguez L. C. Palmer K. L. Frizzo C. P.. (2014). Dicationic imidazolium-based ionic liquids: a new strategy for non-toxic and antimicrobial materials. RSC Adv. 4 (107), 62594–62602. doi: 10.1039/C4RA09906K

[B14] He X. Yang Y. Song H. Wang S. Zhao H. Wei D. (2020). Polyanionic composite membranes based on bacterial cellulose and amino acid for antimicrobial application. ACS Appl. Mater. Interf. 12 (13), 14784–14796. doi: 10.1021/acsami.9b20733 32141282

[B15] Hossain M.I. Samir B. B. El-Harbawi M. Masri A. N. Abdul Mutalib M. I. Hefter G.. (2011). Development of a novel mathematical model using a group contribution method for prediction of ionic liquid toxicities. Chemosphere 85 (6), 990–994. doi: 10.1016/j.chemosphere.2011.06.088 21794892

[B16] Hurley F. H. WIer T. P. (1951). Electrodeposition of metals from fused quaternary ammonium salts. J. Electrochem. Soc. 98 (5), 203. doi: 10.1149/1.2778132

[B17] Ibsen K. N. Ma H. Banerjee A. Tanner E. E. L. Nangia S. Mitragotri S. (2018). Mechanism of antibacterial activity of choline-based ionic liquids (CAGE). ACS Biomater. Sci. Eng. 4 (7), 2370–2379. doi: 10.1021/acsbiomaterials.8b00486 33435102

[B18] Jalal R. Goharshadi E. K. Sajjadi H. Nancarrow P. (2014). Antibacterial activity of short-chained 1-Alkyl-3-Methylimidazolium bis (Trifluoromethylsulfonyl) imide ionic liquids. Phys. Chem. Res. 2 (2), 260–269. doi: 10.22036/pcr.2014.6236

[B19] Jeong S. B. Ko H. S. Heo K. J. Shin J. H. Jung J. H. (2022). Size distribution and concentration of indoor culturable bacterial and fungal bioaerosols. Atmos. Environ. 15, 100182. doi: 10.1016/j.aeaoa.2022.100182

[B20] Kaur N. Fischer M. Kumar S. Gahlay G. K. Scheidt H. A. Mithu V. S. (2021). Role of cationic head-group in cytotoxicity of ionic liquids: probing changes in bilayer architecture using solid-state NMR spectroscopy. J. colloid Interface Sci. 581, 954–963. doi: 10.1016/j.jcis.2020.08.115 32961348

[B21] Kuhn B. L. Kaminski T. F. A. Carvalho Â. R. Fuentefria A. M. Johann B. M. B. C. da Silva E. E.. (2021). Antimicrobial and toxicity evaluation of imidazolium-based dicationic ionic liquids with dicarboxylate anions. Pharmaceutics 13 (5), 639. doi: 10.3390/pharmaceutics13050639 33947119 PMC8145335

[B22] Kuhn B. L. Osmari B. F. Heinen T. M. Bonacorso H. G. Zanatta N. Nielsen S. O.. (2020). Dicationic imidazolium-based dicarboxylate ionic liquids: thermophysical properties and solubility. J. Mol. Liquids 308, 112983. doi: 10.1016/j.molliq.2020.112983

[B23] Łuczak J. Jungnickel C. Lacka I. Stolte S. Hupka J. (2010). Antimicrobial and surface activity of 1-Alkyl-3-Methylimidazolium derivatives. Green Chem. 12 (4), 593–601. doi: 10.1039/b921805j

[B24] Mehta M. J. Kumar A. (2019). Ionic liquid assisted gelatin films: green, UV shielding, antioxidant, and antibacterial food packaging materials. ACS Sustain. Chem. Eng. 7 (9), 8631–8636. doi: 10.1021/acssuschemeng.9b00423

[B25] Mester P. Wagner M. Rossmanith P. (2015). Antimicrobial effects of short chained imidazolium-based ionic liquids-influence of anion chaotropicity. Ecotoxicol. Environ. Saf. 111, 96–101. doi: 10.1016/j.ecoenv.2014.08.032 25450920

[B26] Montalbán M. G. Víllora G. Licence P. (2018). Ecotoxicity assessment of dicationic versus monocationic ionic liquids as a more environmentally friendly alternative. Ecotoxicol. Environ. Saf. 150, 129–135. doi: 10.1016/j.ecoenv.2017.11.073 29272717

[B27] Morawska L. Tang J. W. Bahnfleth W. Bluyssen P. M. Boerstra A. Buonanno G.. (2020). How can airborne transmission of COVID-19 indoors be minimised? Environ. Int. 142, 105832. doi: 10.1016/j.envint.2020.105832 32521345 PMC7250761

[B28] Muñoz-Bonilla A. Fernández-García M. (2018). Poly(Ionic liquid)s as antimicrobial materials. Eur. Polymer J. 105, 135–149. doi: 10.1016/j.eurpolymj.2018.05.027

[B29] Mutuku J. K. Hou W.-C. Chen W.-H. (2020). An overview of experiments and numerical simulations on airflow and aerosols deposition in human airways and the role of bioaerosol motion in COVID-19 transmission. Aerosol Air Qual. Res. 20 (6), 1172–1196. doi: 10.4209/aaqr.2020.04.0185

[B30] Nikfarjam N. Ghomi M. Agarwal T. Hassanpour M. Sharifi E. Khorsandi D.. (2021). Antimicrobial ionic liquid-based materials for biomedical applications. Adv. Funct. Mater. 31 (42), 2104148. doi: 10.1002/adfm.202104148

[B31] Pang L. Q. Zhong L. J. Zhou H. F. Wu X. E. Chen X. D. (2015). Grafting of ionic liquids on stainless steel surface for antibacterial application. Colloids Surf. B: Biointerf. 126, 162–168. doi: 10.1016/j.colsurfb.2014.12.018 25561415

[B32] Piotrowska A. Syguda A. Wyrwas B. Chrzanowski Ł. Heipieper H. J. (2017). Toxicity evaluation of selected ammonium-based ionic liquid forms with MCPP and dicamba moieties on pseudomonas putida. Chemosphere 167, 114–119. doi: 10.1016/j.chemosphere.2016.09.140 27716584

[B33] Qin J. Guo J. Xu Q. Zheng Z. Mao H. Yan F. (2017). Synthesis of pyrrolidinium-type Poly(Ionic liquid) membranes for antibacterial applications. ACS Appl. Mater. Interf. 9 (12), 10504–10511. doi: 10.1021/acsami.7b00387 28272866

[B34] Ratti R. (2014). Ionic liquids: synthesis and applications in catalysis. Adv.Chem. 2014(3), 1–16. doi: 10.1155/2014/729842

[B35] Rezki N. Al-Sodies S. A. Ahmed H. E. A. Ihmaid S. Messali M. Ahmed S.. (2019). A novel dicationic ionic liquids encompassing pyridinium hydrazone-phenoxy conjugates as antimicrobial agents targeting diverse high resistant microbial strains. J. Mol. Liquids 284, 431–444. doi: 10.1016/j.molliq.2019.04.010

[B36] Sahu G. Das M. Sethy C. Wazalwar R. Kundu C. N. Raichur A. M.. (2021). Ionic liquid-assisted fabrication of Poly(Vinyl Alcohol)/Nanosilver/Graphene oxide composites and their Cytotoxicity/Antimicrobial activity. Mater. Chem. Phys. 266, 124524. doi: 10.1016/j.matchemphys.2021.124524

[B37] Singh S. K. Savoy A. W. (2020). Ionic liquids synthesis and applications: an overview. J. Mol. Liquids 297, 112038. doi: 10.1016/j.molliq.2019.112038

[B38] Siopa F. Figueiredo T. Frade R. F. M. Neto I. Meirinhos A. Reis C. P.. (2016). Choline-based ionic liquids: improvement of antimicrobial activity. ChemistrySelect 1 (18), 5909–5916. doi: 10.1002/slct.201600864

[B39] Stolte S. Matzke M. Arning J. Böschen A. Pitner W.-R. Welz-Biermann U.. (2007). Effects of different head groups and functionalised side chains on the aquatic toxicity of ionic liquids. Green Chem. 9 (11), 1170–1179. doi: 10.1039/b711119c

[B40] Vereshchagin A. N. Frolov N. A. Egorova K. S. Seitkalieva M. M. Ananikov V. P. (2021). Quaternary ammonium compounds (QACs) and ionic liquids (ILs) as biocides: from simple antiseptics to tunable antimicrobials. Int. J. Mol. Sci. 22 (13), 6793. doi: 10.3390/ijms22136793 34202677 PMC8268321

[B41] Xiong J. W. Wan M. P. Ng B. F. You S. (2020). Quantification of viable bioaerosol emanation from an ACMV system and its impact on indoor bioaerosol pollution. Aerosol Air Qual. Res. 20 (4), 762–775. doi: 10.4209/aaqr.2019.05.0253

[B42] Yeung K. l. Han W. Song X. Kwan J. K. C. (2020). Ionic liquid-based coating and method of making articles coated with the same. European Patent Office, E.P. Patent No 3 739 006 A1.

[B43] Yoo B. Shah J. K. Zhu Y. Maginn E. J. (2014). Amphiphilic interactions of ionic liquids with lipid biomembranes: a molecular simulation study. Soft Matter 10 (43), 8641–8651. doi: 10.1039/C4SM01528B 25248460

[B44] Zhang Y. Li S. Xu Y. Shi X. Zhang M. Huang Y.. (2022). Engineering of hollow polymeric nanosphere-supported imidazolium-based ionic liquids with enhanced antimicrobial activities. Nano Res. 15 (6), 5556–5568. doi: 10.1007/s12274-022-4160-6

[B45] Zhang J. (. Wei Y. Fang Z. (2019). Ozone pollution: a major health hazard worldwide. Front. Immunol. 10. doi: 10.3389/fimmu.2019.02518 PMC6834528 31736954

[B46] Zheng S. Li W. Ren Y. Liu Z. Zou X. Hu Y.. (2022). Moisture-wicking, breathable, and intrinsically antibacterial electronic skin based on dual-gradient Poly(Ionic liquid) nanofiber membranes. Adv. Mater. 34 (4), 2106570. doi: 10.1002/adma.202106570 34751468

[B47] Zhou Q. Lu X. Zhang S. Guo L. (2014). Physicochemical properties of ionic liquids. Ionic Liquids Further UnCOILed 275–307. doi: 10.1002/9781118839706.ch11

